# Evaluating Enzymatic Productivity—The Missing Link to Enzyme Utility

**DOI:** 10.3390/ijms23136908

**Published:** 2022-06-21

**Authors:** Khawar Sohail Siddiqui, Haluk Ertan, Anne Poljak, Wallace J. Bridge

**Affiliations:** 1 School of Biotechnology and Biomolecular Sciences (BABS), The University of New South Wales (UNSW), Kensington, Sydney, NSW 2052, Australia; wj.bridge@unsw.edu.au; 2 School of Chemical Engineering, The University of New South Wales (UNSW), Kensington, Sydney, NSW 2052, Australia; 3 Department of Molecular Biology and Genetics, Istanbul University, 34134 Istanbul, Turkey; 4 Mark Wainwright Analytical Centre, The University of New South Wales (UNSW), Kensington, Sydney, NSW 2052, Australia; a.poljak@unsw.edu.au

**Keywords:** industrial biotechnology, enzyme, productivity, stability, kinetics, inhibition, biocatalysis, immobilized, chemical modification, genetic modification

## Abstract

Kinetic productivity analysis is critical to the characterization of enzyme catalytic performance and capacity. However, productivity analysis has been largely overlooked in the published literature. Less than 0.01% of studies which report on enzyme characterization present productivity analysis, despite the fact that this is the only measurement method that provides a reliable indicator of potential commercial utility. Here, we argue that reporting productivity data involving native, modified, and immobilized enzymes under different reaction conditions will be of immense value in optimizing enzymatic processes, with a view to accelerating biotechnological applications. With the use of examples from wide-ranging studies, we demonstrate that productivity is a measure of critical importance to the translational and commercial use of enzymes and processes that employ them. We conclude the review by suggesting steps to maximize the productivity of enzyme catalyzed reactions.

## 1. Introduction

Enzymatic productivity is a measure of product formation or substrate disappearance over time, at a prescribed temperature under specified reaction conditions. It is the only measure which reliably summarizes the durability and reaction yield (a measure of the conversion of substrate) of an enzymatic process, factors that are significantly critical to translational or biotechnology applications [1].

As a derivative measure, productivity results from multiple factors, including enzyme activity and half-life, substrate type and concentration, enzyme form and concentration, and exposure time, all of which require a multifactorial experimental design. Therefore, expediency may be the reason that this method of measurement is frequently overlooked in enzyme characterization studies (Table 1).

However, reporting on enzyme activity as a proxy for productivity may not only be misleading, but risks overlooking processes with commercial potential since kinetics based on initial rates ignore the effects that accumulate over long reaction times. For example, lower initial enzyme activity may none-the-less be associated with higher product yield over time, while higher initial enzyme activity may be of short duration with lower ultimate yield [2].

Kinetic productivity analysis can be employed to assess the catalytic capacity of genetically and chemically modified variants [3,4], whole cells [5], the effect of immobilization carriers on productivity [6], difference between isoforms isolated from a range of organisms or tissues, and the effect of reaction solution additives [7].

Here, we suggest that enzyme utility and potential industrial value can only be reliably determined when productivity (reaction yield per unit time, reaction yield per reaction volume per unit time or reaction yield per reaction volume per unit time per enzyme mass) is reported in enzyme characterization studies. To support our case, we provide a few selected examples where kinetic productivity analysis has provided valuable insights into strategies for maximizing enzyme catalyzed reaction yields.

## 2. Typical Enzyme Characterization Methods and Factors That Influence Enzymatic Productivity

Routinely determined conventional parameters in enzyme characterization include activity-based initial reaction rates using Michaelis–Menten kinetics (*V*_max_, *k*_cat_, *K*_m_, *K*_i_) and measures of stability (*T*_opt_, *t*_1/2_, and *T*_m_) (Box 1) [8].

Box 1 **Glossary.**

**Activity-based parameters:***V*_max_: Maximal velocity of enzyme catalyzed reaction; *k*_cat_ (turnover number, *V*_max_/[E]): Number of substrate molecules converted to product by each catalytic site per unit time; *K*_m_: Enzyme-substrate affinity; *k*_cat_/*K*_m_ (specificity) constant or catalytic efficiency: How efficient an enzyme can be on two different substrates; *K*_i_: Enzyme-inhibitor affinity depicting extent and types of inhibition (competitive, non-competitive). **Stability-based parameters:**
*T*_opt_: Optimum temperature of activity; *t*_1/2_: Half-life of irreversible thermal inactivation; *T*_m_: Melting temperature at which 50% of protein structure and/or activity is lost.

**Figure 1:**
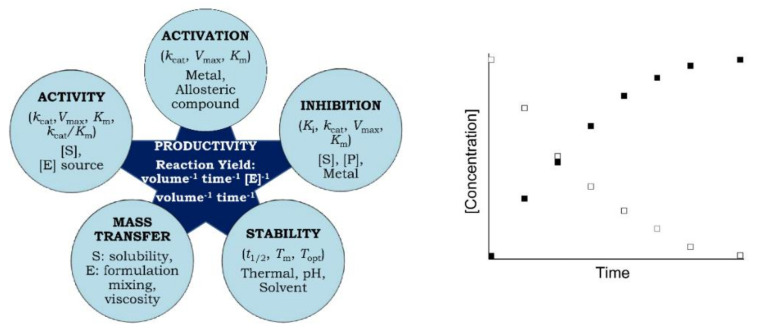
Concept and factors that influence enzymatic productivity. (**Left**): Complex dependency of productivity on the enzyme function, stability, and mass transfer parameters. (**Right**): Hypothetical basic productivity curves showing the formation of product (filled square) or consumption of substrate (open squares) as a function of time under optimal reaction conditions. [E]: Enzyme; [S]: Substrate; [P]: Product concentrations.

However, since the time required to measure them is considerably shorter than the time required to prepare a productivity curve, these metrics do not predict how the enzyme will perform with extended and repeated use under specific reaction conditions, such as temperature, pH, additives, and substrate and enzyme concentrations. Enzyme reaction rates over time are continuously influenced by rapidly changing reaction compositions and conditions, such as decreasing substrates and increasing product concentrations as well as their effect on enzyme activation and inhibition. Additionally, productivity is affected by the progressive unfolding of the enzyme over the course of the reaction and modification of key amino acids. These can all affect final reaction yields, a particularly important factor in commercial bioprocesses (Figure 1, left) [9].

Enzyme kinetics play a key role in productivity under certain conditions. For example, *K*_m_ and *k*_cat_/*K*_m_ (Box 1) are important intrinsic parameters, but under reaction conditions where [S] >> *K*_m_, catalytic efficiency is not pertinent. However, for artificial substrates where the *K*_m_ may be high, the specificity constant turns into the more relevant variable. Furthermore, neither *k*_cat_ nor *k*_cat_/*K*_m_ indicates whether the enzyme will last on the time scales (hours/days) of productivity analysis, as these are determined from initial reaction rates (a few minutes).

Temperature impacts enzyme activity in a complex manner (Figure 1, left). For example, temperature optimum (*T*_opt_) is influenced by the duration of the enzyme assay. Additionally, the melting temperature (*T*_m_) is based on the structural unfolding (denaturation) of the enzyme and depends on the method used to detect this property. Far-UV circular dichroism is used to follow secondary structure, whereas intrinsic fluorescence, near-UV CD, and differential scanning calorimetry are employed to follow tertiary structure unfolding. In all these methods, the extent of stability (*T*_m_) depends upon the scan rate. On the other hand, *t*_1/2_ measures the activity regained after the enzyme has been cooled and refolded and can impact productivity. The increase in temperature enhances enzyme activity up to a certain limit. However, further increase in temperature might result in decreased productivity due to enzyme inactivation [10]. Moreover, elevated temperatures have been shown to lower reaction rates for some enzymes due to irreversible unfolding arising from temperature-dependent interconversion of active and inactive forms of the enzyme [11]. For other enzymes exposed to elevated temperatures, the active site has been shown to initially unfold (local unfolding) prior to an overall structural unfolding (global unfolding). In addition to temperature, enzymes are destabilized by solvents, salts, pH, and other environmental factors. Further complications arise due to the activity–stability trade-off, which implies that highly stable enzymes are less active, whereas highly active enzymes, such as cold-adapted enzymes, are more thermolabile [3,7].

Another important factor related to productivity stems from the mass transfer limitation that can influence the rate of supply of the substrate to the enzyme active site (Figure 1, left). Mass transfer depends on several factors, including the type of substrate (simple vs. polymeric), the enzyme form, such as heterogeneous formulation (whole cells and immobilized), viscosity of the reaction medium that can change during the reaction with the product formation, reaction components which are not properly mixed, and how the substrate is dosed.

## 3. How Productivity Curves Can Be Prepared

Productivity curves can be readily generated by incubating equal or known amounts of enzymes (from two or more different organisms, isoforms or wild-type and genetically and chemically modified, immobilized enzymes or whole cells containing enzyme/s) with substrate/s in the presence or absence of additives at a specific temperature under optimum reaction conditions. Aliquots are withdrawn at regular intervals throughout the reaction, which is eventually quenched by any method that denatures or inactivates the enzyme. The formation of product or the disappearance of substrate (no matter which is more convenient) is then plotted as a function of time after correcting for any non-enzymatic reaction (Figure 1, right) [9]. The quantification of product or substrate can be followed by any suitable measure, such as change in absorbance, fluorescence or viscosity, radiometric, manometric, polarimetric, chromatographic, electrophoretic, electrochemical or mass spectrometric methods depending on the availability of equipment and consumables, convenience, simplicity, speed, safety, and cost [12]. Productivity is generally expressed as volumetric productivity (amount of product formed per reaction volume per unit time) or specific volumetric productivity (volumetric productivity per mg or g of enzyme) [13]. Whereas enzyme assays are based on the initial rate of substrate utilization in the absence of product formation and are usually completed within minutes, the duration of productivity analysis can extend to hours with significant depletion in substrate concentration and accumulation of product.

Lately, Woodley et al. [2] have proposed more informative productivity plots compared with the basic plots commonly used (Figure 1, right). In these modified plots, the X-axis of basic productivity plot (Figure 1, right) is normalized by multiplying it by [E]. In this productivity plot (time x [E] vs. conversion), limitations due to enzyme kinetics, stability, and mass transfer can be identified if the plots do not superimpose. Further information can be extracted through varying the substrate concentration by plotting time x [E]/[S] vs. conversion at constant [E] concentrations. As with [E], varying [S] can change the time required to attain the provided productivity [2]. These modified plots are discussed in detail in Section 7.2 and Section 7.3, along with examples.

## 4. Why Productivity Analysis Is Necessary?

The attainment of high volumetric productivity or specific volumetric productivity in an enzyme catalyzed reaction involves the complex interplay between several parameters depicted in Figure 1 (left). For example, immobilization generally reduces activity due to the suboptimal substrate supply to the enzyme active site (mass transfer limitation), but may increase activity as a result of enhanced stability and/or relieved inhibition. Kinetic characterization of enzymes using productivity curves overcomes the limitation of using *k*_cat_, *K*_m_, *k*_cat_/*K*_m_, *K*_i_, *T*_opt_, *t*_1/2_, and *T*_m_ as the sole criteria in evaluating enzyme performance, resulting from the significant scarcity of productivity data in the literature (Table 1) [14]. Productivity is the one indicator of an enzyme’s potential commercial utility, as it encompasses both the catalytic activity, protein stability, and mass transfer limitations associated with an enzyme reaction over extended and repeated use. A critical reason for maximizing productivity is the fact that costs of enzymes are generally a central component of the industrial/commercial processes that utilize them, and are also a major total cost contributor [1]. By maximizing productivity, costs can be minimized, and process efficiency and durability can be maximized. Productivity plots normalized on the basis of [E] and [S] concentrations can be used to identify underlying constraints that can guide the optimization of reaction processes at an industrial scale.

## 5. Significance and Applications of Productivity Analysis

Productivity curves monitor yields throughout a reaction process under specific conditions (pH, temperature, ionic strength, substrate and enzyme concentrations). Therefore, the amount of reaction product at the end of an extended period of time is dependent upon the irreversible inactivation of enzyme due to thermal unfolding and/or substrate/product inhibition. In this way, different forms of an enzyme, such as native vs. modified, soluble vs. immobilized [9,14,15,16] can be evaluated and more efficient enzymes can be identified and compared across studies. Moreover, productivity can be maximized in the presence of an additive [17] or by varying other reaction conditions, such as ionic strength, pH, and [S] and [E] concentrations [14].

### 5.1. Higher Productivity Due to Chemical Modification

**Figure 2:**
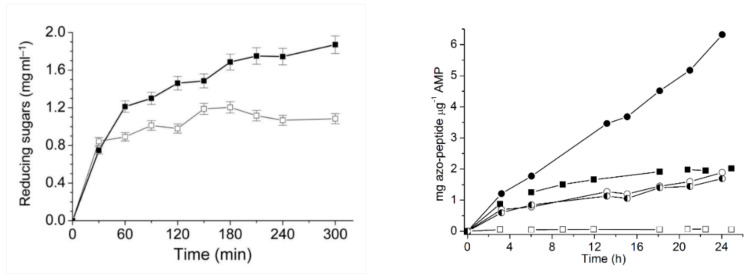
Productivity curves for alkaline metalloprotease, α-amylase (**left**), and AMP (**right**). Starch hydrolysis by α-amylase at 60 °C as determined by the formation of reducing sugars. Sodium acetate/acetic acid buffer (50 mM, pH 5) and enzyme (0.32 μg mL^−1^) were added to the starch solution (6% *w/v*), to initiate the reaction. Open symbols: Unmodified α-amylase; filled symbols: Modified α-amylase (reproduced from [16] with permission from Oxford University Press). Protein hydrolysis by native, EDTA-treated, and Ca^2+^-treated alkaline metalloproteases (AMP) at different temperatures. Formed azo-peptide (mg µg^−1^AMP) was measured using azocasein as substrate (1% *w/v*) in 0.1 M MES-NaOH buffer, pH 6.5 over a period of 24 h. Circles: Reaction performed at 40 °C; squares: Reaction performed at 48 °C. Open symbols: EDTA-treated AMP; half-filled symbols: Native enzyme; filled symbols: +5 mM Ca^2+^ (reproduced from [17] with permission from Elsevier).

The formation of reducing sugars from starch hydrolysis by unmodified and modified forms of α-amylase over 300 min at 60 °C is shown in Figure 2 (left). Following 5 h at 60 °C, the modified enzyme hydrolyzed nearly twice the quantity of starch as compared with the unmodified enzyme. Although the modified α-amylase possessed only 60% of the intrinsic activity (*k*_cat_) of the unmodified enzyme, the higher productivity attained by the modified enzyme was due to its relatively high thermostability over the duration of the hydrolysis reaction [16].

Higher productivities of cold-adapted and mesophilic lipases have been achieved by chemical modification of enzymes using benzoic anhydride, Ficoll, and 5 kDa of PEG. Higher productivities of all lipases were due to their higher protein stability and resistance to thermal unfolding at higher temperature. Moreover, the modified lipases retained better activity in paint emulsions after 20 weeks of incubation at 25 °C, indicating that they may have a potentially superior value for industrial applications [18].

An interesting case of higher productivity of Savinase at low temperatures has been reported [15]. Savinase is a serine protease with extensive biotechnological applications in a broad range of industries, including surfactant products, textile, food and animal feed, leather, photographic, cosmetic, environmental remediation, and pharmaceutical industries. A bulky modifier (dextran, 70 kDa), when attached to Savinase, prevented the enzyme from allosteric uncompetitive substrate inhibition with a concomitant 5-fold increased reaction yield (azopeptides) relative to the unmodified enzyme (5 °C after 50 h). Conversely, at higher temperature (55 °C), the modified protease showed 10-fold less yield relative to the unmodified enzyme, as the second molecule of the substrate (azocasein) was unable to bind to the allosteric site due to steric hindrance by the modifier, and thus was unable to stabilize the modified enzyme. In this example, the higher productivity of the modified enzyme at low temperatures was due to an increase in the activity (relieving the enzyme of substrate inhibition), whereas the modified enzyme showed lower productivity at higher temperature due to thermostability [15].

### 5.2. Higher Productivity via Genetically Modified Organisms or Enzymes

#### Production of Guanidinoacetate by Genetically Modified *Bacillus subtilis* Whole Cell Catalysis

Guanidinoacetate (GAA) is used as feed/food additive in pharmaceutical industry. The bacterium was genetically modified by introducing the arginine:glycine amidinotransferase (AGAT, EC 2.1.4.1) gene that catalyzes the following reaction:

L-arginine + glycine → L-ornithine + guanidinoacetate

The gene expression of AGAT was optimized and the genes responsible for glycine and arginine degradation were knocked out. Additionally, the ornithine pathway was optimized to reduce the arginine waste and prevent product inhibition of AGAT by ornithine. With the use of 20 g L^−1^ of glycine and arginine at 30 °C and 220 rpm shaking, a volumetric productivity of 4.26 g/L was achieved in 20 h with a maximum productivity of 1.58 g/L/h. The productivity (g L^−1^ h^−1^) decreased with time up to 20 h, accounting for cell death and degradation [5].

The volumetric productivity of halohydrin dehalogenase, which is commercially used in the synthesis of a cholesterol lowering drug, atorvastatin (Lipitor) was improved by 4000-fold with genetic modifications compared with the unmodified enzyme at 40 °C, pH 7.3, and substrate concentration of 130 g L^−1^ [9].

### 5.3. Higher Productivity Due to Additives

Similarly, productivity curves of a cold-adapted alkaline metalloprotease (AMP) showed that the total product yield after 24 h (Figure 2, right) was higher at 40 °C compared with 48 °C. This was probably due to the rapid unfolding of cold-adapted AMP at higher temperature. Furthermore, there was an approximately 3-fold increase in the amount of azopeptides formed at 40 °C in the presence of Ca^2+^ compared with the native and EDTA-treated enzymes (Figure 2, right). The reason for the higher productivity was found to be the higher intrinsic activity (*k*_cat_), as well as higher thermal stability (*t*_1/2_, *T*_m_, *T*_opt_) of the protease in the presence of Ca^2+^ [17].

Theanine is an FDA approved health drink additive. The addition of monovalent cations (Na^+^, Cs^+^) in the presence of Mg^2+^ to γ-glutamyl transpeptidase (GGT) from *Bacillus licheniformis* enhanced the production of theanine by 15% relative to the absence of metal additives [19]. The productivity analysis was carried out in a reaction mixture containing 25 μg mL^−1^ GGT, 250 mM L-Gln as donor, 600 mM ethylamine as acceptor, ±200 mM Na^+^ or ±Cs^+^ as well as ±200 mM Mg^2+^ in 50 mM borate buffer (pH 10.5) at 37 °C for 4 h. The reason for the higher productivity in the presence of monovalent cations was found to be an increase in both the *V*_max_ (from 17 to 28 µM min^−1^) and thermostability (*t*_1/2_ at 60 °C, from 16 to 74 min^−1^).

### 5.4. Higher Productivity Due to Immobilization

**Figure 3:**
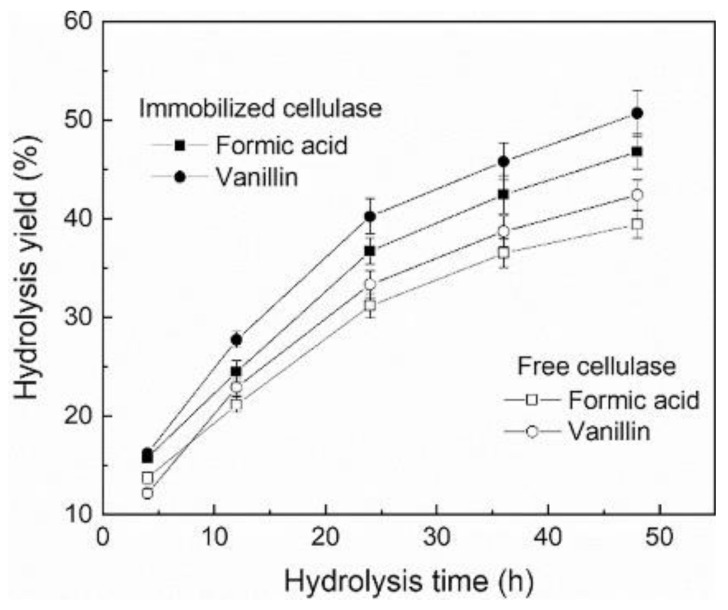
Enzymatic hydrolysis of microcrystalline cellulose in the presence of inhibitors (5 g L^−1^) at 50 °C using free and immobilized enzyme on magnetic MOF nanoparticles. The product (glucose) was determined by HPLC using ion-exclusion chromatography (reproduced from [21] with permission from Elsevier).

To improve the economics of the reaction, enzymes have been immobilized on magnetic nanomaterials to simplify the recycling process by overcoming fouling issues, which commonly arise with the more traditional use of membrane separation of the enzyme from the reaction liquor [20]. However, all forms of immobilization techniques can positively or negatively impact an enzyme’s reaction kinetics and overall productivity. An example of where magnetic immobilization has been shown to provide productivity improvement potential [21] is in the manufacture of industrial ethanol from lignocellulosic biomass [22]. During the cellulase and β-glucosidase catalyzed pretreatment of lignocellulose, vanillin and formic acids are formed as by-products. Both of these are inhibitory to the enzymes’ activities. In an investigation to determine the potential of immobilized cellulases, the enzyme was attached to a magnetic Fe_3_O_4_-metal organic framework. Then, the activity profiles of the immobilized and free enzymes were compared in the hydrolysis of microcrystalline cellulose (MCC, 2% *w/v*) in the presence of 5 g L^−1^ of both vanillin and formic acids. The reactions were conducted at 50 °C with 150 rpm mixing and operated for 24 h. It was observed that the immobilized cellulase provided about 20% higher hydrolysis yield relative to the free enzyme. In conclusion, the immobilization had enhanced productivity by relieving the inhibition exerted by vanillin and formic acids. The immobilized enzyme stability was explored using the hydrolysis reaction cycle for 24 h by 5 times on 5% MCC. Following each of the five reactions, the immobilized enzyme was isolated from the liquor using a magnet prior to washing, and then added to the fresh substrate of the next reaction cycle. Following the completion of the five cycles, the magnetic cellulase was shown to have retained 70% of its initial productivity (Figure 3) [21].

The productivity analysis of single vs. enzyme mixtures (endo-, exo-cellulase, and β-glucosidase) aimed at synergistic activation [23] and native vs. pretreated lignocellulosic biomass, aimed at reducing the recalcitrant nature of substrates, is expected to play a critical role in optimizing cost-effective enzymatic hydrolysis of lignocellulosic biomass for biofuel production [24].

Similarly, β-galactosidase from *Kluyveromyces lactis* immobilized on aldehyde-activated or glutaraldehyde-activated agarose had higher productivities (99% hydrolysis) than soluble β-galactosidase (90%). This increase in productivity was achieved due to a reduction in non-competitive product inhibition by glucose [25]. Moreover, the same enzyme was immobilized via cross-linked enzyme aggregation (CLEA) and showed 4-fold higher lactose hydrolysis after 2 h at room temperature with retention of all initial activity after 10 cycles of reusage [26].

### 5.5. GGT Catalyzed Processes for the Synthesis of Pharmaceuticals

γ-Glutamyl cysteine (GGC) is currently marketed as a precursor to boost the synthesis of glutathione in the body [27,28]. GGT catalyzes the transfer of γ-glutamyl group to various acceptors and is a key enzyme for the commercial production of GGC and theanine (see Section 5.3). Glutathione and its precursors are potential novel adjunct therapies to improve the survival of patients with COVID-19 pneumonia. The mechanism of action is to inhibit or ameliorate the cytokine storm, which is induced by viral mediated cellular glutathione depletion during infection [29]. The industrial processes for the manufacture of GGC and theanine are impacted by the high price of the purified GGT enzymes, which can be extracted from various organisms. Therefore, it is critical for manufacturers to consider the use of productivity curves to maximize the synthesis of GGC and theanine under optimum reaction conditions and in the most cost-effective manner. Under optimized conditions (37 °C; pH 9; *Bacillus licheniformis* GGT, 1 U ml^−1^), 85–87% synthesis of theanine was achieved from ethylamine (600 mM) and glutamine (80 mM) within 4 h. Immobilized GGT did not show any enhancement in productivity compared with the free enzyme, as the increase in the thermostability of the immobilized enzyme was accompanied by a 60% decrease in its specific activity. However, the immobilized enzyme could be reused 5 times with only 10% loss in activity up to 3 cycles, thereby minimizing manufacturing costs [28]. Recently, GGT from the same organism showed 15% enhancement in theanine productivity in the presence of metal ion additives (Na^+^ or Cs^+^ + Mg^2+^) due to the simultaneous increase in activity and stability (see Section 5.3) [19]. It would be interesting to investigate the productivity of theanine with immobilized GGT in the presence of metal ion additives by combining the advantages of immobilization (recycling of GGT) and additives (enhanced productivity). Other examples show that the comparison of GGT productivity curves across sources (mammalian, bacterial, and fungal) can facilitate the identification of factors, which maximize process yields, and these may well differ in their transpeptidase:hydrolysis ratio using various substrates (donor and acceptor) [30,31].

### 5.6. Use of Inclusion Bodies for Manufacturing Fabric

Recently, the applications of catalytically active inclusion bodies (CatIBs) for the economical production of highly stable and recyclable enzymes have provided an alternative to immobilized enzymes. CatIB variants of lysine decarboxylase (LD) were generated in *Escherichia coli* by fusing five different types of aggregation-inducing tag (AIT) to the enzyme via flexible S/G or rigid P/T linkers for the production of 1,5-diaminopentane (DAP). In industry, DAP is used as a precursor for creating polyamide in waterproof abrasion resistant fabrics. CatIBs incorporating LD-(PT)_17_ linker-L_6_KD AIT provided the highest volumetric productivity (457 g L^−1^ d^−1^) and specific volumetric productivity (256 g L^−1^ d^−1^ g CatIB^−1^), which was determined within 20 min at 30 °C. By contrast, the wild-type enzyme without any linker and AIT produced only half the yield (volumetric productivity, 219 g L^−1^ d^−1^; specific volumetric productivity, 106 g L^−1^ d^−1^ g CatIB^−1^) relative to the CatIBs [13].

### 5.7. An Example Where Productivity Analysis Was Overlooked

From the examples discussed above, it is apparent that ignoring productivity analysis in enzyme studies can result in overlooking key insights and losing potential commercial opportunities. A case in point is a recent study on levansucrase, which produces fructooligosaccharides and polymers with wide applications in food and medical biotechnology [32]. A double variant with higher thermostability and lower activity relative to the native enzyme was constructed with a view to enhance the yield of the levan-type fructooligosaccharides. However, the lack of productivity curves precluded drawing a confident conclusion, in which the engineered variant provides better product yield than the native enzyme.

## 6. Basis for Higher Productivity

Although the productivity analysis will reveal which form of enzyme (native vs. modified, soluble vs. immobilized) or enzyme source provides the higher yield under optimal reaction conditions, the analysis does not inform us on the basis of its higher productivity. Higher productivity can be due to many factors, such as enhanced thermal stability, higher intrinsic activity, reduced inhibition of the enzyme or mass transfer. To dissect the basis for the higher yield of an enzyme, its Michaelis–Menten kinetics (*V*_max_/*k*_cat_, *K*_i_, *k*_cat_/*K*_m_), and thermostability assays (*t*_1/2_, *T*_opt_, *T*_m_) should be carried out. Table 2 lists the variables that can affect enhanced productivity.

## 7. Optimization of Parameters for Enhancing Productivity

Once the most efficient form of an enzyme has been identified, the reaction conditions (temperature, pH, substrate and enzyme concentrations, additives, etc.) should be optimized to maximize the reaction yield in the shortest time. If the basis of the effect of increased productivity is related to kinetic or stability improvements, then genetic or chemical modification can be considered for improving *k*_cat_, *K*_m_, *t*_1/2_ of inactivation and/or *T*_opt_. If the substrate and product are unfavorably impacting productivity, their concentrations can be controlled via substrate feeding or product removal. Problems related to mass transfer can be overcome by reactor design and configuration (substrate introduction and transport, cosolvent selection). Another key factor that impacts productivity is enzyme formulation and choice between whole cell biocatalysts, crude or purified enzyme, soluble or immobilized enzyme [2].

### 7.1. Optimization of Temperature

#### 7.1.1. Lipase Mediated Hydrolysis

**Figure 4:**
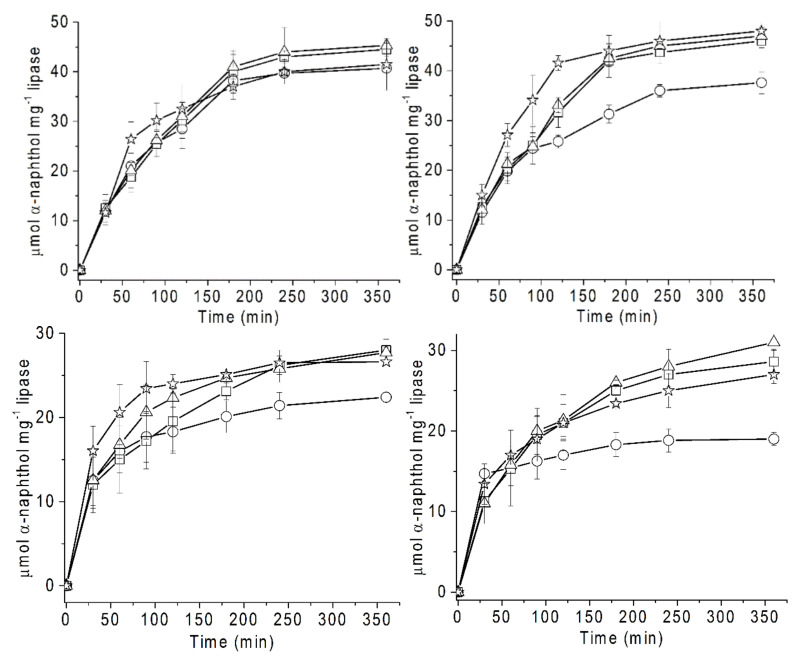
Productivity of unmodified and modified lipases from thermally-adapted lipases at different temperatures. Above, two figures: 50 °C; below, two figures: 75 °C. Left, two figures: Cold-adapted lipase from *Candida antarctica*; right, two figures: Mesophilic *Humicola lanuginose*. Circle: Native; square: Benzoic anhydride-modified enzymes; triangle: Polyethylene glycol-modified enzymes and star Ficoll-modified enzymes. All experiments were performed in triplicate and error bars are shown for each data point (reproduced from [18] with permission from Elsevier).

A case in point is the productivity involving native and chemically modified lipases from cold-adapted, *Candida antarctica* and mesophilic, *Humicola lanuginose* at two different temperatures (Figure 4). The productivity of modified variants of lipases was higher than the native enzyme at both temperatures, with the difference as more pronounced at higher temperature (Figure 4, figures below) and for mesophilic lipases (Figure 4, right figures). The productivity of all lipases, which was higher at 50 °C compared with 75 °C, implied that the higher temperature inactivated the enzyme during the course of productivity analysis (Figure 4, figures below). This shows the significance of optimization of temperature for maximizing the yield. Recently, the addition of metal ion additives increased the *t*_1/2_ of thermal inactivation of GGT by 5-fold, resulting in increased productivity due to the ability of the enzyme to better resist the thermal unfolding over an extended period (see Section 5.3) [19].

#### 7.1.2. Designer Cellulosome Mediated Deconstruction of Cellulose

In this groundbreaking study, cellulosome was designed by combining thermostable scaffoldin subunit with thermostable endoglucanase, exoglucanase, and β-glucosidase enzymes via genetic modifications. Readers are referred to the review by [35] for the architecture of the cellulosome. The thermostable cellulosome was used to hydrolyze microcrystalline cellulose into glucose at various temperatures (50–80 °C). The optimization showed that the thermostable designer cellulosome provided higher productivity up to 96 h at 60 °C compared with mesophilic designer cellulosome, thermostable free enzyme mixture, and mesophilic free enzymes. Notably, thermostable designer cellulosome provided 1.7-fold greater productivity than mesophilic designer cellulosome. This study is a fine demonstration of the fact that mere improvements in the activity and/or stability of an enzyme do not assure its suitability for use in the catalytic conversion. The actual improvement can only be tested via productivity analysis, which takes into account the synergy between various enzyme components, as well as the complex interplay between stability, activity, and substrate/product inhibition and mass transfer limitations [34].

### 7.2. Optimization of Substrate Concentration

Commercial immobilized penicillin acylase was assayed using 7-amino-3 desacetoxycephalosporanic acid (7-ADCA) as substrate and product formation of cephalexin was measured at 14 °C. In the presence of 30% (*v*/*v*) ethylene glycol, the yield of cephalexin increased by approximately 30-fold when the substrate concentration was increased from 30 mM (productivity: 10 mM/h) to 200 mM (productivity: 298 mM/h) [14]. Subsequent work demonstrated that productivity could be further improved to 384 mM h^−1^ in the absence of ethylene glycol with an average activity loss of less than 2% per batch, enabling the process to be carried out for about 60 batches before the need for enzyme replacement [36]. This shows the significance of optimization of substrate concentration for maximizing the yield.

**Figure 5:**
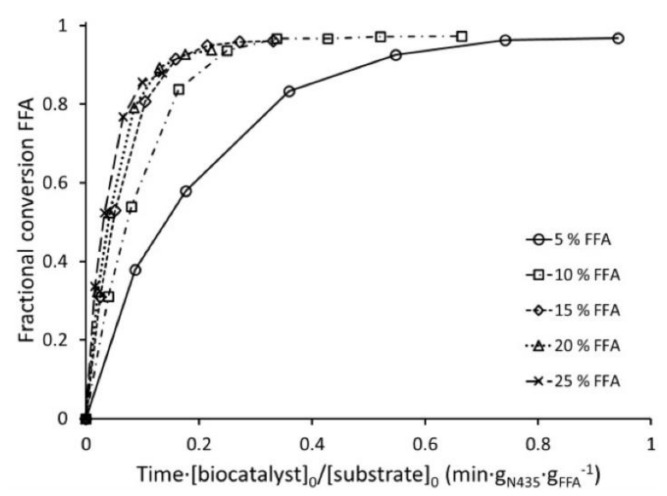
Effect of oleic acid (free fatty acids (FFA)) concentrations (*v*/*v*) in the esterification of methanol in vegetable oil at 45 °C using 5% (*w*/*v*) CalB immobilized lipase (reproduced from [2] with permission from Elsevier).

The esterification of oleic acid with methanol for the production of biodiesel using cold-adapted immobilized lipase from *Candida antarctica* (CalB) was analyzed by constructing productivity curves (time x [E]/[S] vs. conversion) at different FFA concentrations, while the concentration of the second substrate (methanol) remained constant at 4% (*v*/*v*). The reaction was completed within an hour, and productivity increased with the substrate concentration, indicating that CalB has higher *K*_m_ for oleic acid (Figure 5). Parallel experiments showed that productivity is independent of enzyme concentration although higher concentrations of methanol decreased productivity. This suggests that methanol acted as a competitive inhibitor for oleic acid. Both the competitive inhibition by methanol and high *K*_m_ for oleic acid were later confirmed by kinetic experiments [2]. It would have been very interesting if the productivity experiments (time × [E]/[S] vs. conversion) would have been evaluated at two additional temperatures (above and below 45 °C) to dissect the balance between temperature-associated increase in enzyme activity and inactivation due to lipase unfolding.

### 7.3. Optimization Involving Enzyme Concentration Due to The Mass Transfer Limitation

Generally, productivity increases linearly with an increase in enzyme concentration. However, due to mass transfer restraints, the reaction rate will be reduced after a certain critical enzyme concentration has been reached. Secondly, there is a limit as to the amount of enzyme that can be added to the reactor. These considerations are particularly important for immobilized enzymes. For soluble enzymes, increasing the concentration can enhance the rate of biocatalyst inactivation, thereby decreasing overall productivity over time. When biocatalytic conversion of glucose (200 mM) to gluconic acid and H_2_O_2_ was carried out in the presence of 50 and 100 mg L^−1^ glucose oxidase and air at 25 °C (500 rpm at 1 vvm air), it was found that productivity (time × [E] vs. conversion) was not doubled as expected. The reason for this discrepancy was found to be the mass transfer limitation of the second substrate (O_2_) supply [2].

### 7.4. Optimization Involving Multiple Enzymes

**Figure 6:**
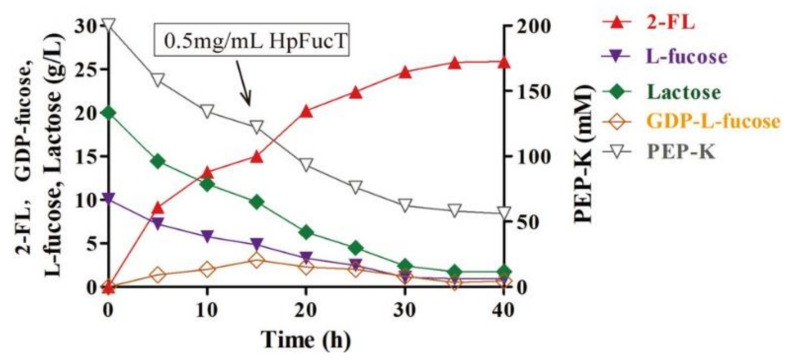
Productivity curve for the synthesis of 2-Fl (red triangle) in the presence of multiple enzymes. The second addition of fucosyltransferase is indicated by an arrow. The depletion of L-fucose (blue triangle), lactose (green diamond), PEP-K (open triangle), and the formation of GDP-fucose intermediate (open diamond) during the reaction are shown. Only L-fucose and lactose as starting substrates are shown, whereas GTP and ATP are constantly recycled by pyruvate kinase during the reaction, resulting in reduced feedback inhibition and savings due to expensive cofactors (reproduced from [37] with permission from The American Chemical Society).

A very good example of meticulous optimization to maximize productivity involved the application of an in vitro multi-enzyme cascade catalysis system (MCCS). During MCCS, four different enzymes (bifunctional L-fucokinase/GDP-L-fucose phosphorylase, α-1,2-fucosyltransferase, and pyruvate kinase) were employed for the synthesis of 2-fucosyllactose in a series of complex reactions. Nutraceutically important, 2-fucosyllactose is used as an additive in infant milk, and is currently extracted from human breast milk or chemically synthesized in a tedious process. Following the careful optimization of enzyme source and their concentration and reaction conditions, the temperature, pH, GTP:ATP ratio, phosphoenolpyruvate potassium (PEP-K) salt, PEP-K concentrations, and buffers provided a volumetric productivity of 0.73 and 0.67 g L^−1^ h^−1^ for α-1,2-fucosyltransferase from *Helicobacter pylori* and *Thermosynechococcus* *elongatus,* respectively in Tris/HCl, pH 7.5 buffer at 35 °C, and 1:2 ATP/GTP M ratio containing 200 mM PEP-K (Figure 6). When the activity and stability of the rate-limiting α-1,2-fucosyltransferase from *H. pylori* and *T. elongatus* were determined, it was found that *H. pylori* enzyme had higher *k*_cat_ (22 min^−1^) and *k*_cat_/*K*_m_ (39 min^−1^ mM^−1^) than *T. elongatus* (*k*_cat_: 3 min^−1^; *k*_cat_/*K*_m_: 12 min^−1^ mM^−1^), but lower stability (*T*_m_: 42 vs. 48 °C). This implies that the higher activity and specificity constant of α-1,2-fucosyltransferase from *H. pylori* was the cause for its higher yield [37]. For example, other processes that employ multiple enzyme mixtures are exo-/endo-cellulases and/or β-glucosidase for the hydrolysis of lignocellulosic biomass into sugars [24] or ethanol [38] and deconstruction of polyethylene terephthalate (PET) synthetic plastic by the sequential action of cutinase enzymes, PETase and mono(2-hydroxyethyl) terephthalate (MHETase) into terephthalic acid and ethylene glycol [39]. Compared with separate enzymes, the depolymerization of PET is enhanced when PETase and MHETase are combined via linkers into a single chimeric enzyme, thus increasing productivity by synergistic activation.

### 7.5. Optimization Involving Reaction Conditions

GGT catalyzes various transpeptidase reactions depending on donors (glutamine, glutamyl ethylester, etc.) and acceptors (amino acids, small peptides, ethylamine, etc.). The yield of the final product critically depends on the reaction mode, which includes the donor and acceptor nature, concentrations, and their ratio. γ-Glutamylvalylglycine, which is known as *kokumi,* enhances the taste and texture of the food and is synthesized by reacting glutamine (donor) with Val-Gly dipeptide (acceptor) in the presence of *E. coli* GGT. The highest productivity of γ-glutamylvalylglycine was obtained at 20 mM Gln and 100 mM Val-Gly in the presence of 5 % NaCl and 60 mM MgCl_2_, pH 8 at 37 °C. The optimal Gln:Val-Gly ratio was found to be 1:5, which was key to maximizing the yield. The high yield could be accomplished by increasing the concentration of both substrates or by supplying Gln as it is consumed. Increasing the substrate, such as the acceptor concentration, is not possible due to solubility limitations. The optimal donor:acceptor ratio (1:5) was maintained by feeding Gln every 5 h up to 20 h into the reactor, which led to 1.7 times better yield relative to the yield without periodic Gln feeding (Table 2) [33].

**Figure 7:**
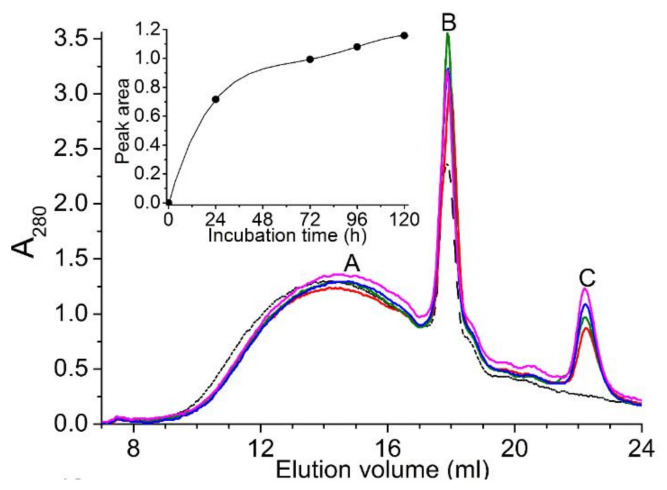
Size-exclusion chromatography profiles of humic acid (HA) degradation by VP, where both MnP and LiP were equally active. Peak A: High molecular weight HA; Peak B: Intermediate molecular weight HA; Peak C: Low molecular weight degradation products of HA. 0 h: Dotted line; 24 h, red; 72 h, green; 96 h, blue; 120 h, magenta. Inset: Productivity of HA degradation as determined by the area under peak C (reproduced from [41] with permission from Elsevier).

Hybrid VP consisting of manganese peroxidase (MnP) and lignin peroxidase (LiP) activities was used to degrade humic acid (HA) under reaction conditions (0.1 M tartaric acid/NaOH, pH 4 buffer containing 0.22 mM MnSO_4_), where both MnP and LiP were equally active [40]. The molecular weights of VP catalyzed HA were determined using size-exclusion chromatography and confirmed by electrospray ionization mass spectrometry (ESI-MS). The high molecular weight HA Peak A was degraded into lower molecular weight degradation products (Peak C) via an intermediate Peak B (Figure 7). The inset in Figure 7 shows the productivity (formation of lower molecular weight compounds) with an extended time. Moreover, the degradation was confirmed by ESI-MS, which showed that various products differed by 44 Da in consistency with the C_2_H_4_O functional group [41]. This example shows the fine tuning of reaction conditions in a way that both enzyme activities (MnP and LiP) within a single enzyme were equally active to synergistically degrade HA (Figure 7).

## 8. Reproducibility and Data Deposition

There are no critical reproducibility issues with productivity analysis except for the routine of replicate aliquots, which should be followed (Figure 3 and Figure 4). For better reproducibility, it is vital to minimize evaporation during productivity analysis, particularly at higher temperatures and for assays that require longer duration (Figure 3).

To date, there is no repository for productivity data deposition. BRENDA enzyme database is the most comprehensive database for functional, molecular, and structural information on enzymes [42]. However, BRENDA currently lacks data on volumetric or specific volumetric productivity. The BRENDA curators agree that productivity data will be a useful addition to the BRENDA functional enzyme properties (personal communication). Addition of the new information is pending consideration of optimal data integration and formatting.

## 9. Conclusions and Future Outlook

Due to the practical significance of enzyme catalyzed reactions in biochemical manufacturing, productivity curves are suggested to be generated under various conditions (pH, temperature, [S] and [E] concentrations) to assist in the optimization of reaction yields (Figure 1, left; Table 2) and minimize operational costs.

**Figure 8:**
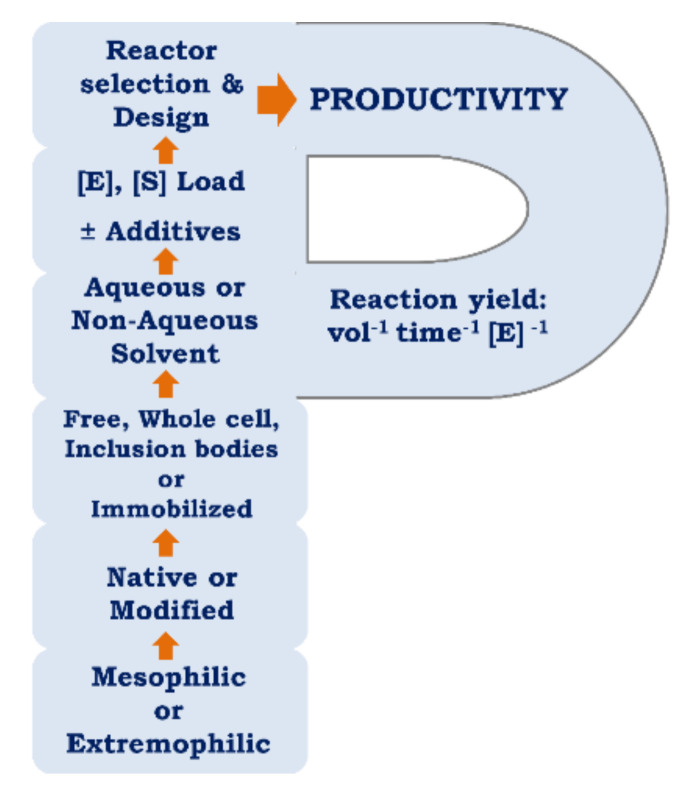
Hierarchical strategy to maximize reaction productivity based on a prioritized optimization of enzymes, solvents, additives, and enzyme reactors. Bottom to top: Step 1., Preferred enzyme activity profile depicted by the direction of arrows: Source options include mesophiles or extremophiles that offer enzymes with activities that are psychrophilic, thermophilic, acidophilic, alkaliphilic, halophilic, piezophilic, and/or organic-solvent tolerant [43]. Step 2., Enzyme source and optimization: Unmodified, genetically [8,9,32] or chemically modified [15,16] variants. Step 3., Enzyme formulations: Free, inclusion body [13] or immobilized [20,22,38]. Step 4., Reaction solvent: Carrying aqueous or non-aqueous ((e.g., organic solvent [8,14], ionic liquid or super-critical CO_2_ [44]). Step 5., Varying [E] and [S] concentrations [2]. Additives or co-factors that improve yields, activity, and productivity [17,44]. Step 6., Reaction mode and reactor design: For example, batch, continuous, solid state or plug flow [45].

Productivity maximization of enzyme catalyzed manufacturing process also requires optimization of other key reaction parameters [8]. These involve the selection of appropriate enzyme sources and formulations, yield improving enzyme modifications, choice of reaction solvents and additives, and enzyme reactor designs and modes of operation. This should all be considered in the initial planning of the process, with subsequent optimizations prioritized in the following hierarchy: enzyme activity and form, reaction conditions, and reactor design (Figure 8).

Productivity is a measure of critical importance to the translational and commercial use of enzymes and the process that utilizes them. Consequently, overlooking this method of measurement during enzyme characterization risks missing not only important data, but may also ignore important cost-effective processes and commercial opportunities [10].

## Figures and Tables

**Table 1:** Statistics of articles published on enzyme properties, which exclude or include enzymatic productivity.

Search Words with Boolean Operators ^a, b^		
	NOT Productivity	AND Productivity ^c, d^
Enzyme AND characterization	61,817	70 (430)
Enzyme AND mutagenesis	18,095	11 (156)
Enzyme AND chemical modification	1693	3 (9)
Enzyme AND immobilization	20,308	132 (390)
Total	101,913	216 (985)

^a^ The Web of Science Core Collection was used for the search, and the analysis date range was 2000–2021. ^b^ Searched within title, abstract, and authors’ keywords. All research article types were included, except review articles, proceeding papers, book chapters, and editorial materials. ^c^ Relevance of all articles was manually checked to exclude non-enzymatic productivity articles. ^d^ The values in parenthesis are raw article numbers before manual elimination of articles not related to enzyme productivity.

**Table 2:** Activity, stability, inhibition or substrate concentration: Basis for enhanced productivity.

Enzyme	Modification/ Additive	Activity	Stability	[Substrate]	Inhibition	Reference
α-amylase	Native vs. CM	Dec.	Incr.	NA	NA	[16]
Lipase Lipase	Native vs. CM Im	Dec. Nd	Incr. Nd	NA 5–25%	NA Nd	[18] [2]
Savinase	Native vs. CM	Incr.	Dec.	NA	Dec.	[15]
β-galactosidase	Native vs. Im	Dec.	Incr.	NA	Dec.	[25]
Metalloprotease	Native vs. +Ca^2+^	Incr.	Incr.	NA	NA	[17]
Penicillin acylase	Im vs. Im	NA	NA	Incr. 30–200 mM	NA	[14]
*GGT (Bl) GGT (Bl) GGT (*E.coli*)	Native vs. Im ±Additives Native	Dec. Incr. Nd	Incr. Incr. Nd	NA NA [donor:acceptor]	NA NA NA	[27] [19] [33]
Cellulase Cellulosome	Native vs. Im ^@^ GM: Meso- vs. thermophilic	Incr. Var.	Incr. Incr.	NA NA	Dec. NA	[21] [34]

CM: Chemically modified; Im: Immobilized; both GGT (γ-glutamyl transpeptidase); Bl: *B. licheniformis*; * no net increase in productivity of free vs. immobilized enzyme; ^@^: Immobilized on Fe_3_O_4_-metal organic framework nanomaterial; NA: Not applicable; Nd: Not determined; GM: Genetically modified; Dec.: Decreased; Incr.: Increased; Var.: Variable.

## Data Availability

Not applicable.
